# Supplementary figures and images for: Building Resilience and Competence in Bachelor Nursing Students: A Narrative Review Based on Social Cognitive Theory
Source: Nurs Rep. 2025 Jul 11;15(7):253. doi: 10.3390/nursrep15070253 (PMC12298541; doi:10.3390/nursrep15070253)

**Figure S1. PRISMA Flowchart.**

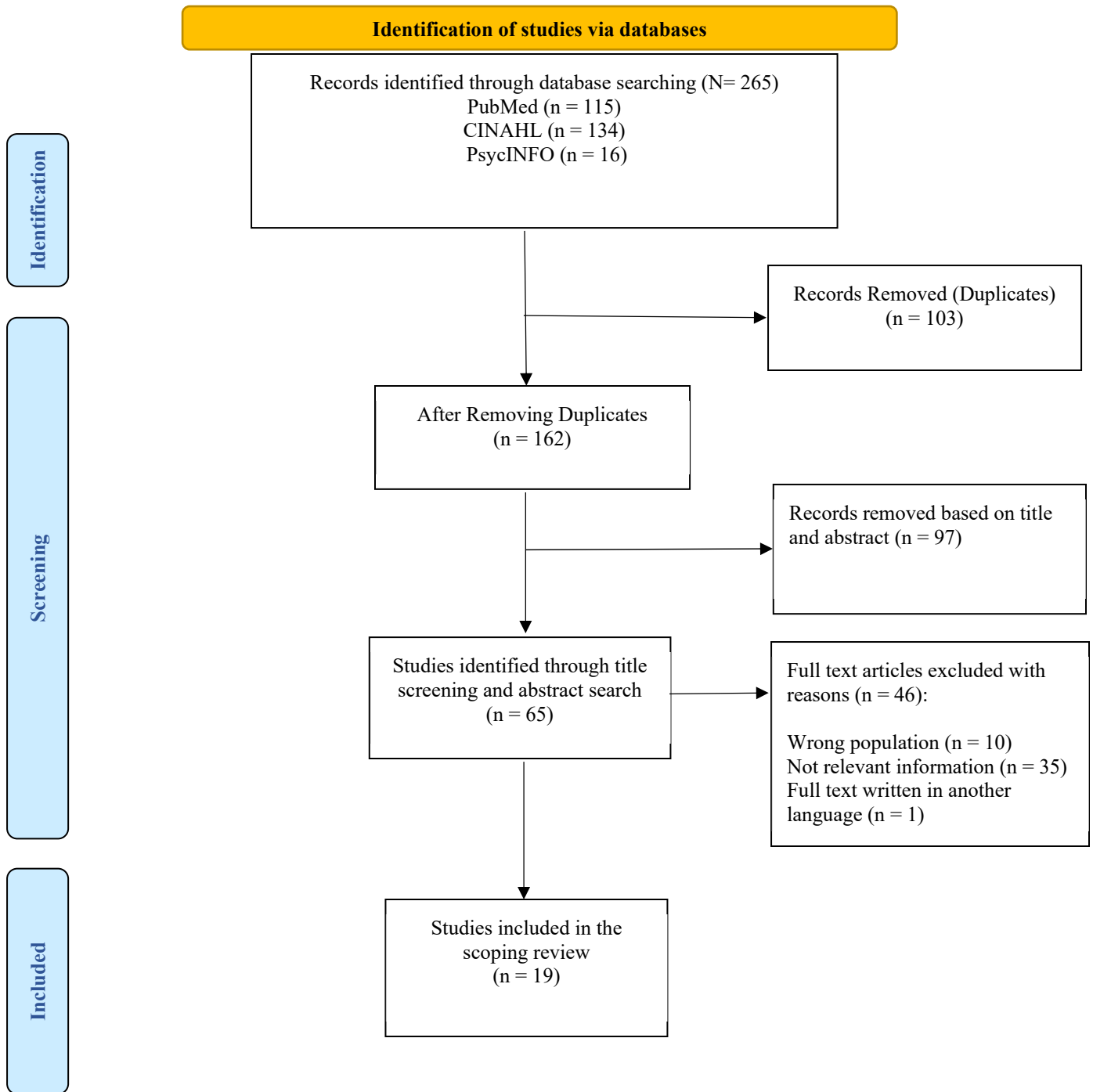

Supplement: Supplementary file 1 [file nursrep-15-00253-s001.zip › nursrep-3736966-supplementary.pdf]
